# Optimizing Stem Cells Mobilization Strategies to Ameliorate Patient Outcomes: A Review of Guide- lines and Recommendations

**Published:** 2017-01-01

**Authors:** Saeed Mohammadi, Ashraf Malek Mohammadi, Mohsen Nikbakht, Amir Hossein Norooznezhad, Kamran Alimoghaddam, Ardeshir Ghavamzadeh

**Affiliations:** Hematology-Oncology and Stem Cell Transplantation Research Center, Tehran University of Medical Sciences, Tehran, Iran

**Keywords:** Stem cell, Mobilization, Peripheral blood, Transplantation

## Abstract

Peripheral blood stem cell transplantation (PBSCT) is an effective treatment for hematological malignancies. Mobilization of peripheral blood progenitor cells performs in different ways among transplantation centers. Forceful mobilization schedules are comprised of growth factor alone, chemotherapy along with growth factor and also, a newly combination of novel agent such as plerixafor with any approach. With the appearance of numerous modifications in stem cell mobilization field over the past decade and advent of novel stem cell mobilization techniques, it seems to be necessary to review recent publications about stem cell mobilization strategies to respond above cited issues. Relevant literature was identified by a PubMed search (1996–2016) of English-language literature using the terms mobilization, Allogeneic Stem Cells Transplantation, Autologous Stem Cells Transplantation and technical aspects of apheresis. Although many institutions have established their own procedures to improve stem cell mobilization success rates accompanying cost-effectiveness considerations, an optimal stem cell mobilization regimen and methods have not been well-defined, yet. Practical guidelines are required to address critical clinical issues including proper growth factor, the most Impressive chemotherapy and its dosage and appropriate time for leukapheresis initiation. Hence, based on literature, we prepared practical guidelines in this review.

## Introduction

 Hematopoietic Stem cells transplantation (HSCT) is become a curative option for patients who suffer from hematological malignancies. 1^,^^2^  The usage of both autologous and allogeneic HSCT for adults and pediatric has exceedingly increased, over the past several decades. Small amounts of hematopoietic stem cells (HSCs) are able to circulate in Peripheral blood (PB).^   3 ^ So, HSCs mobilization from bone marrow (BM) to PB and their collection can be crucial element of HSCT programs. 4^,^^5^  Despite the vast using of peripheral stem cells transplantation (PBSCT) as therapeutic strategy, it is difficult to achieve a consensus about its parameters. These parameters are type of growth factor and its optimal dosage, effectiveness type of chemotherapy and its dosage and how to predict poor mobilize patients and which time is best to initiate leukapheresis.^        6 ^ Nowadays, most transplantation institutions have adjusted own strategies according to their priorities and resource availabilities. Therefore, there are not any standard identical approaches. Hence, this paper aims to review current literature and guide lines on mobilization strategies to underscore the importance of mentioned problems.

## Methods

 Mobilization guidelines for autologous and allogeneic transplantation were obtained by the way of literature search. Extracted information about mobilization schedules, laboratory monitoring protocols and technical aspects of apheresis for adults and pediatrics are main foundations of presented guide lines in our review.

## Results

**CSF dose recommendation for Allogeneic Transplantation in Adults**   ^7^^-^^12^ 

**1-   The recommended dose for sibling donors**

- 5 µg/kg G-CSF twice per day as a split dose or 10 µg/kg/day as a single dose is advised.

- Using higher split dose (12 µg/kg twice/day) results in higher collection yields with shorter collection time.

**2-   The recommended dose for unrelated donors**

- G-CSF is administered for 4 or 5 consecutive days at a dose of 10 µg/kg daily.

- During the PBSCs collection, the total processed blood volume (TPBV) does not be exceeding of 24 liters and it should be collected during 1 or 2 consecutive days.

**Target Stem Cells dose for Allogeneic Transplantation in Adults** ^14^^-^^19^

**1-   Transplantation from sibling donors**

- The common accepted cell dose is 2×10^6^ CD34‏ cells/kg at least.^5^^,^^12^^,^^13^ Successful engraftment has reported at dose as low as 0.75×10^6^ CD34‏ cells/kg, whereas neutrophil and particularly platelet engraftments were delayed. Hence, more transfusion of blood components is required.

- Based on available data, CD34‏ cells dose between 4 and 5×10^6^ CD34‏ cells/kg seems to be most acceptable amount for allogeneic transplantation in adults. Several studies have shown that higher doses of CD34‏ cells infusion are associated with faster engraftment.

- Any count more than 8×10^6^ CD 34 cells/kg could enhance risk of extensive chronic GVHD without any improvement in survival of patients.

**2-   Transplantation from unrelated donors**

- Any count more than 9×10^6^ CD 34 cells/kg did not result in any further survival benefits. Likewise, higher cell doses are not associated with worsening GVHD.

**G-CSF dose recommendation for Allogeneic Transplantation in Pediatric** ^20^^-^^22^ 

- The most common approach makes use of G-CSF is 10 µg/kg as a single or two semi-doses per day.

**Target Stem Cells dose for Allogeneic Transplantation in Pediatric** ^23^^-^^25^ 

- Minimum amount of collected cells are reported 2.4×10^6^ CD34‏ cells/kg for allogeneic transplantation in pediatric.

- Higher CD34‏ cell counts (>4-5×10^6^) have been associated with faster engraftment while no impact on overall survival or the risk for developing GVHD was observed.

**Figure 1:**
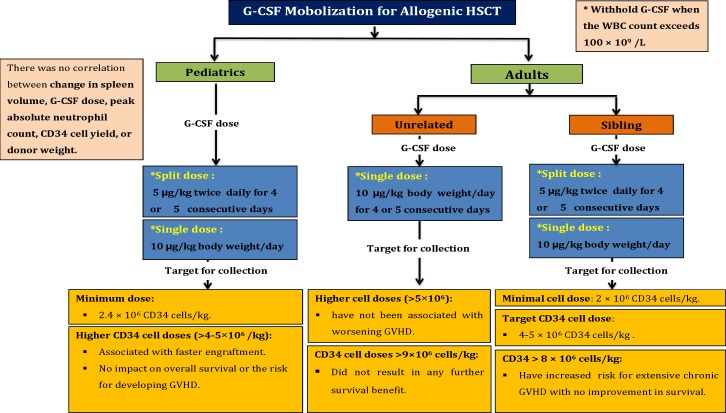
A summary of stem cells mobilization strategies and target cells dose for allogeneic stem cells transplantation

A summary of stem cells mobilization strategies and target cells dose for allogeneic stem cells transplantation is shown in Figure 1.

**Mobilization Strategies for Autologous Transplantation in Adults**

**1) G-CSF alone strategy** ^26^^-^^28^ 

**1A) G-CSF alone strategy utilization for Multiple Myeloma (MM) patients**

- In these subjected patients with not more than 1 previous line of therapy or negative history of previous treatment with melphalan or>4 cycles of lenalidomide, the best choice is just G-CSF with following schedule:

- A daily single dose of 10-16 µg/kg G-CSF with subcutaneous injection is most common. No advantages have been observed by split dosing of G-CSF. There is evidence on efficacy of 12 µg Pegfilgrastim as a single subcutaneous dose in these patients.

Optimal harvest is possible when G-CSF is given 3 hours before apheresis versus administration was performed on the evening before apheresis. For acquire proper consequence, leukapheresis should be beginning on the fifth day.

- Although growth factor alone is often adequate for patients with early-stage of MM, it is often suboptimal for them at late stage.
- In patients with more than 1 previous line of therapy or history of previously treated with melphalan or >4 cycles of lenalidomide: In such patients, PB CD34‏ cells count monitoring with preemptive Plerixafor will lead to successful collection in the vast majority of patients.

**1B) G-CSF alone and/or Plerixafor strategies utilization about None-Hodgkin Lymphoma (NHL)**

- Although persistent mobilization with G-CSF alone with doses of 10-16 µg/kg/day associated with higher incidence of failure rates in some patients or which thing is suboptimal but it may be an option owing to low toxicity and ease of scheduling. So that, this strategy will not suitable for patients with high risk of mobilization failure.
- PB CD34‏ counts monitoring with preemptive plerixafor will lead to successful collection in the vast majority of patients.

**2) Chemotherapy plus growth factor mobilization strategies**   ^29^^-^^32^ 

**G-CSF and chemotherapy dose elements**

- In this strategy, 5 to 10 µg/kg G-CSF as a single daily dose or pegfilgrastim (5 μg/kg/day) as a single administration can be used at the beginning of leukapheresis when peripheral blood CD34‏ cell counts or WBC count is adequate. In addition to, Lenograstim 150μg/m^2^/day (equivalent of 5 μg/kg/day subcutaneously) is advised to choose.
- Cyclophosphamide (3-7 gr/m^2^) or etoposide is commonly used for mobilization which results in higher collection yields during fewer days of apheresis than mobilization with growth factor alone. These benefits occur at the expense of increased hospitalizations for neutropenic fever, which occur in a substantial portion of patients. Current data do not declare that the concern which mobilization regimens include etoposide promote secondary malignancies.

**2A) Chemotherapy plus growth factor mobilization strategy in Multiple Myeloma patients:** High-dose cyclophosphamide + G-CSF are probably the most commonly used chemo mobilization strategy. Some studies also suggest that etoposide-based mobilization approaches can be considered as alternative choice.

**2B) Chemotherapy plus growth factor mobilization strategy in Lymphoma patients: **Chemotherapy + G-CSF as part of disease specific induction and salvage regimens have always regarded the preferred method. Such approaches can eliminate to require additional chemo-mobilizations or steady-state mobilizations before auto-HSCT in these heavily treated patients. Further, it is more effective than cyclophosphamide-based chemo-mobilization.

**Optimal G-CSF Dose for Initial Mobilization in Pediatric Autologous Transplantation** ^33^^,^^34^ 

- G-CSF (10 µg/kg/day or 12 µg/kg given twice per day) with leukapheresis beginning on the fifth day of G-CSF could result in successful mobilization in one day.

**Target Stem Cells Dose for Autologous Transplantation in Adults and Pediatric** ^35^^-^^39^ 

- 2×10^6^ CD34‏ cells/kg for single transplant has generally been accepted as a safe minimum count. Lower counts will concomitant the risk of delayed neutrophil and platelet engraftment.
- 5×10^6^ CD34 cells/kg has been accepted as suitable optimal collected cells for successful transplantation. Higher CD34‏ cell doses(>6×10^6^/kg) have been associated with faster hematopoietic recovery, more robust long-term platelet recovery, reduced blood transfusion requirements and beside improved overall survival. However, there was no significant difference at the time of platelet recovery to 20×10^9^/L.

**Special Considerations for Obese Patients**  ^40^^,^^41^ 

- Either single daily dose (14 µg/kg/day) or split dose was suggested (2×7 µg/kg/day) for obese patients.
- Patients were stratified according to body mass index (25<BMI>25). In patients with BMI>25 kg/m^2^, once-daily dosing resulted in a higher CD34‏ cells yield.

**Figure 2:**
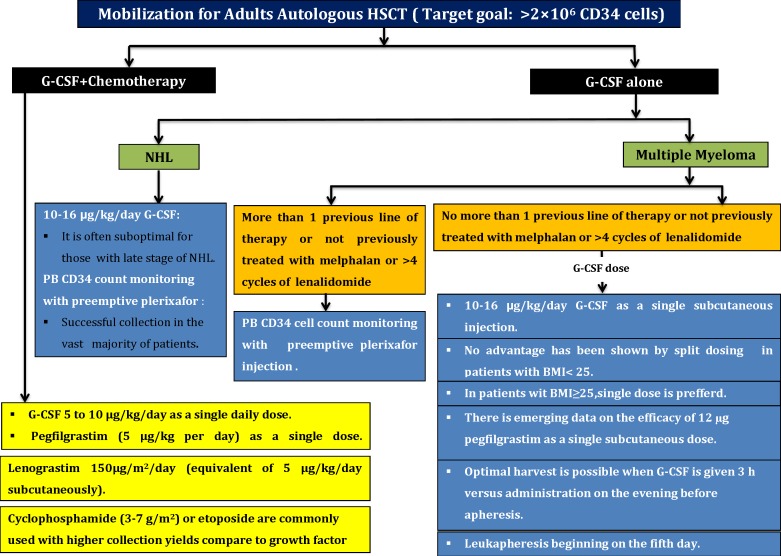
A summary of stem cells mobilization strategy and target cells dose for autologous stem cells transplantation in adults

A summary of stem cells mobilization strategy and target cells dose for autologous stem cells transplantation in adults is shown in Figure 2.

**Apheresis Procedure in Pediatric Patients with Low Weight** ^20^^,^^42^

- Patients with low weight should have characterized by hemoglobin at least level of 12 g/dL. Otherwise, it should be reached to mentioned level by RBC transfusion.
- In children who weigh less than 20 kg, the apheresis machine should be primed with RBCs and/or human albumin to lower the extracorporeal volume.
- In severe thrombocytopenia situation, platelet transfusion should be considered to achieve platelet count above 40×109/L in order to prevent bleeding complications.

**Figure 3:**
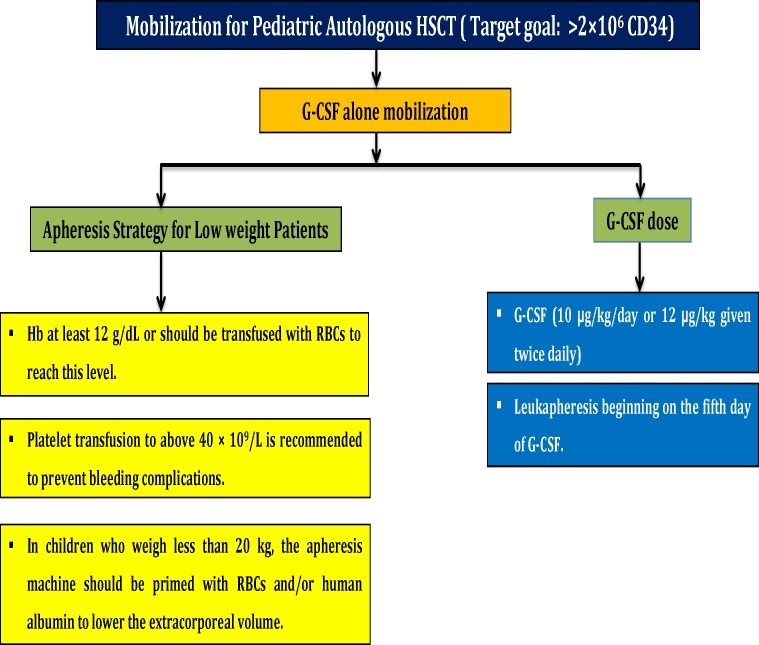
A summary of stem cells mobilization and apheresis strategies and target cells dose for autologous stem cells transplantation in pediatrics

A summary of stem cells mobilization and apheresis strategies and target cells dose for autologous stem cells transplantation in pediatrics is shown in Figure 3.

**Monitoring Peripheral Blood CD34** **‏** **cell Counts in Adults and Pediatrics Autologous Transplantation**    ^45^^-^^50^ 

**1-   Legitimate reasons to G-CSF alone strategy selection**

- CD34‏ cell counts peak in blood between the fourth to sixth days of therapy was observed. In this schedule, CD34‏ cells monitoring should begin on either day 4 or 5.^29^^,^^32^^,^^43^^-^^45^

**2-   Plerixafor plus G-CSF mobilization strategy:**

- CD34‏ cell counts were checked on days 4 and 5 of G-CSF administration.

**3-   Chemo mobilize strategy option**

- CD34‏ counts generally begin 8 to 10 days after chemotherapy administrations.

**Golden Time to Leukapheresis Initiation**   ^44^^,^^45^^,^^51^ 

In chemo-mobilization strategy, the start of leukapheresis is commonly determined by threshold of CD34‏ cell counts. There is no consensus on optimal threshold; therefore, institutional practice or institutional local instructions have varied has varied from minimal CD34‏ counts of 5 to 20/µL.

**Prediction of High Risk Patients for Stem Cell Mobilization Failure**   ^51^^-^^57^ 

**Prediction of mobilization after chemotherapy**

**1-Proven poor mobilizes definition**

- If the patient received 10 μg/kg G-CSF alone or ⩾5 μg/kg G-CSF after chemotherapy but circulating CD34+ cells peak is remained <20/μL for up to 6 days after mobilization will defined as proven poor mobilisers
- Furthermore, if the CD34 cells yield <2.0×10^6^ /kg in⩽3 apheresis up to 20 days after chemo-mobilization regiment, it will define in this category.

**2-Predicted poor mobilisers**

- Meet two main following criteria:

Advanced disease (⩾2 lines of chemotherapy).

- Extensive BM involvement or cellularity <30% at the time of mobilization.

**3-Other criteria are comprised**

- More than 60 years old

- Multiple chemotherapy regimens
- Prior exposure to alkylating medications
- Prior radiation
- Prior treatment with lenalidomide, fludarabine and other purine analogues and also melphalan
- Platelet count below 100 ×10^9^/L
- Previous auto-HSCT
- Low Hb level and WBC count before mobilization

**Poor mobilization prediction in Multiple Myeloma patients**

- <12 months of therapy and a platelet count > 200×10^9^/L, it is possible to obtain ≥4×10^6^ CD34+ cells/kg in a single apheresis.
- Patient over 70 years old with >12 months of prior therapy and platelets < 200 × 10^9^/L; however, were considered as poor mobilizers

**Prediction of Mobilization Failure According to CD34** **‏** **Cells yield**  ^44^^,^^45^^,^^58^ 

**1-Prior to apheresis** ^11^^–^^19^

- **Borderline’ poor mobilisers:** CD34 cells/μL at maximum stimulation will detected in PB and it could be yielded 1.5-2×10^6^/kg CD34 cells after apheresis.

- **Relative’ poor mobilisers:** 6–10 CD34 cells/μL at maximum stimulation will detected in PB and it could be yielded <1×10^6^/kg CD34 cells after apheresis.
- **Absolute’ poor mobilizers** **:** ≤5 CD34 cells/μL at maximum stimulation will detected in PB and it could be yielded about 0.75×10^6^/kg CD34 cells after apheresis.

**2-After apheresis**

- **Optimal collection:** is defined as ≥5 ×10^6^ CD34‏ cells/kg yield.
- **Low collection:** ≥2 to <5 × 10^6^ CD34‏ cells/kg.

- **Poor collection:** <2 × 10^6^ CD34‏ cells/kg.
- **Failed collection:** apheresis is impossible because of low peripheral CD34‏ cell counts.

**Figure 4:**
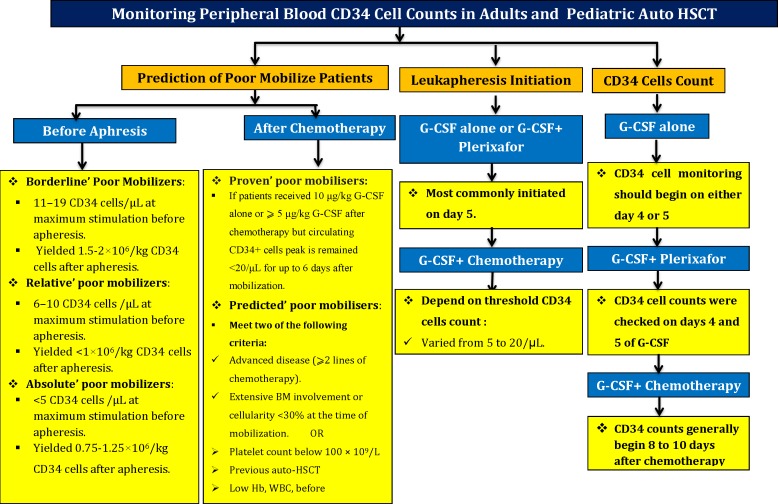
A summary of strategies for prediction of poor mobilize patients in autologous stem cells transplantation

A summary of strategies for prediction of poor mobilize patients in autologous stem cells transplantation is shown in Figure 4.

**Strategies for Poor Mobilizes** ^59^^-^^65^

**1-Border Line Poor Mobilize**

- Large-volume leukopheresis: In this strategy, 4.0–5.3 folds of the patient’s total blood volume should be considered as a TPBV. No difference has observed in CD34+ cells viability compared with normal-volume apheresis (2.7–3.5 folds of the patient’s total blood volume). Relatively poor mobilisers or patients with high individual CD34+ cell collection goal (⩾3 transplants) have indication for large volume leukopheresis.
- Plerixafor addition to standard mobilization strategies will be considered for patients who still mobilize poorly with larger-volume approaches.
- A rest period of 2 to 4 weeks will be considered for patients who fail initial mobilization attempt.
- Plerixafor plus G-CSF (without chemotherapy).
- The addition of Plerixafor to G-CSF alone or G-CSF + chemotherapy: This strategy lead to mobilize more CD34+ cells, increase the proportion of more-primitive HSC subsets. Likewise, positive correlation between the number of re-infused NK cells and early absolute lymphocyte recovery after auto-HSCT has been reported.
- Preemptive intervention with Plerixafor.

**Relatively Poor Mobilize and Poor Mobilize**

- Preemptive use of plerixafor should be considered.

**Figure 5:**
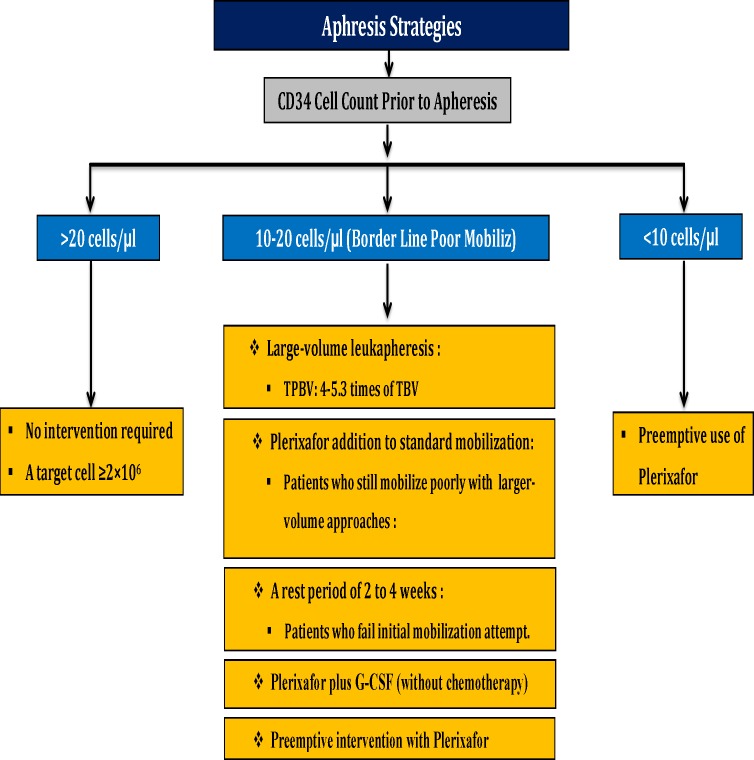
A summary of apheresis and mobilization strategies based on CD34 cell count prior to apheresis for poor mobilize patients in autologous stem cells transplantation.

A summary of apheresis and mobilization strategies based on CD34 cell count prior to apheresis for poor mobilize patients in autologous stem cells transplantation is shown in Figure 5.

- **Threshold of Leukocytosis for Growth Factor Held **   ^66^^-^^68^ 

- In one-third patients, WBC counts developed exceeding 50 × 10^9^/L, while less than 1% of patients had WBC counts exceeding 75×10^9^/L.
- There was no splenic rupture or thrombosis.

- During G-CSF mobilization, significant increase in spleen size was observed. The median spleen volume increased by 1.47-folds on first day of leukapheresis but declined to near pretreatment size after 7 days of leukapheresis. In only 9% of patients splenic volumes increase into more than 2-folds.

- There was no correlation between change in spleen volume, G-CSF dose, absolute neutrophil count peak, CD34‏ cells yield, or donor weight.
- Despite the lack of documentations to support the association between hematological parameters and splenic enlargement or risk of splenic rupture, current data declared that G-CSF and Plerixafor administration should withhold when the WBC count exceeds 100 × 10^9^ and exceeds 75×10^9^/L, respectively.

## CONCLUSION

 PBSC mobilization can be enhanced with a proper approach in allogeneic and autologous HSCT. In autologous HSCT, depended on the patient’s disease and treatment protocol the results of stem cell collection will be different. A low CD34+ cell count in PB before apheresis is a key predictor factor for poor PBSC collection. Hence, enumeration of CD34+ cell prior apheresis may appraisal the risk for poor PBSC collection and could permit suitable intervention to overcome of mobilization failure.
